# Skeletal and dentoalveolar effects of class II malocclusion treatment using bi-maxillary skeletal anchorage: a systematic review

**DOI:** 10.1186/s12903-022-02363-3

**Published:** 2022-08-10

**Authors:** Maged S. Alhammadi, Amal Abdulsalam A. Qasem, Aisha Mohammed S. Yamani, Rawan Duhduh A. Duhduh, Rahaf T. Alshahrani, Esam Halboub, Abeer A. Almashraqi

**Affiliations:** 1 Orthodontics and Dentofacial Orthopedics, Department of Preventive Dental Sciences, College of Dentistry, Jazan University, Jazan, Saudi Arabia; 2 Department of Orthodontics, Pedodontics and Preventive Dentistry, Faculty of Dentistry, Sanaʼa University, Sanaʼa, Republic of Yemen; 3 Internship Program, College of Dentistry, Jazan University, Jazan, Saudi Arabia; 4 Department of Maxillofacial Surgery and Diagnostic Sciences, College of Dentistry, Jazan University, Jazan, Saudi Arabia; 5 Department of Pre-Clinical Oral Health Sciences, College of Dental Medicine, QU Health, Qatar University, Doha, Qatar

**Keywords:** Bi-maxillary skeletal anchorage, Miniplate, Miniscrew, Class II malocclusions, Skeletal effect

## Abstract

**Background:**

The goal of this systematic review was to assess the available evidence regarding the skeletal and dentoalveolar effects of bi-maxillary skeletal anchorage devices (BMSADs) used in treating growing class II malocclusion patients.

**Methods:**

A comprehensive search was conducted on PubMed, Scopus, Science Direct, Web of Science, Cochrane, and LILACS up to November 2021, which was augmented by a manual search. The studies included were clinical trials (RCTs) and/or follow-up observational studies (retrospective and prospective). The outcomes of interest were the skeletal, dentoalveolar, and occlusal treatment-induced changes obtained from pre- and post-cephalometric measurements. The risks of bias of the included studies were assessed using an assessment tool from previous publications.

**Results:**

Out of 742 screened articles, only 4 were eligible and thus included in the qualitative synthesis. They showed a moderate overall risk of bias. The results are presented as mean changes in both the study and control groups. All studies reported retrusion of the maxillary base and advancement of the mandible (meaning reduced ANB angle). Three of the included studies reported an increase in the vertical jaw relation, which was contrary to what the fourth study reported. Three studies reported an increase in the maxillary incisors’ inclination or position, while one study reported their retroclination. Proclination of the mandibular incisors happened in two studies, whereas the other two studies reported retroclination. The overjet was reduced in all included studies.

**Conclusion:**

Apart from the protrusive effects on the mandible, retrusive effects on the maxilla, and the consequent reduction of the overjet, BMSADs results in inconsistent skeletal and dentoalveolar effects. However, the current evidence is limited due to the variability in the biomechanics of the intermaxillary components, type of anchorage, and comparable groups in the included studies. Further RCTs with more standardized methodologies are highly encouraged.

**Clinical relevance:**

BMSADs (using miniscrews or miniplates on both jaws) induces more skeletal than dentoalveolar effects. However, this must be practiced with caution, based on the benefit to risk (surgical insertion) ratio, and the limited evidence available in hand so far.

*Registration* The protocol for this systematic review was registered at the International Prospective Register of Systematic Reviews (PROSPERO, No.: CRD42020199601).

## Background

### Rationale

Malocclusion is the third most common oral health problem following caries and periodontal diseases [1]. Worldwide, class II malocclusion represents almost one-third of recorded malocclusions and is more prevalent in Caucasians than other races, reaching up to 63% in Belgium [2]. Accordingly, in daily dental practice, approximately one-third of patients seeking orthodontic treatment are class II malocclusion patients [3]. This type of malocclusion is attributed to different factors, and most studies have attributed it to mandibular deficiency in the majority of cases, which necessitates the use of mandibular advancement appliances [4–6].

Treatment of skeletal class II malocclusion during the preadolescent stage can be achieved by growth modification, which involves inhibition of maxillary growth and/or stimulation of the mandibular growth [4, 6, 7]. For this purpose, orthopaedic appliances such as extra-oral headgear, removable appliances, or fixed functional appliances (FFAs) can be used [8]. Removable functional appliances are bulky and hence annoying to children, and there is contradictory evidence regarding their therapeutic efficiency. Some researchers have reported favourable treatment effects on mandibular growth, such as effective condylar growth [9–11] and increased mandibular length [12–14]. However, other researchers found no significant effect [15, 16].

There is similar controversy regarding the effect of these appliances on the maxillary jaw. While some studies concluded that there is a restricting effect [17, 18], other studies argued against it [19]. The exact opposite applies in regard to their dentoalveolar effects: There is agreement that these appliances result in proclination of mandibular incisors and retroclination of maxillary incisors [20]. Similarly, FFAs cause more dentoalveolar effects than the skeletal ones [21–23]. Overall, the evidence from systematic reviews and meta-analyses confirms that neither removable appliances nor FFAs produce pure skeletal changes; instead, their effects are almost dentoalveolar [20, 24–27].

With the development and introduction of the skeletal anchorage devices in orthodontics, the limitations of conventional orthopaedic and orthodontic mechanics have been overcome. Skeletal anchorage devices were used on a single jaw to counteract the effect of FFAs on the mandibular incisors [28–31]. Recently, they have been used on both jaws, aided by inter-maxillary protracting force, to maximize the skeletal effect of the planned orthopaedic treatment [7, 32–34]. The biomechanical point of view behind using the skeleltal anchorage is to tranfer the applied force to the underlying bone either to prevent the un-wanted effect of the direct force application to the fixed functional appliances or aiming to tranfer the force directly to the jaw bone to produce the the required growth modification.

A few systematic reviews and meta-analyses have assessed the skeletal and dentoalveolar effects of using skeletal anchorage on a single jaw to support the mandibular advancement appliances [35–37]. New methods have been suggested to attach miniscrews or miniplates on both jaws aiming ultimately to induce pure skeletal effects. However, there has not been a single systematic review so far evaluated the evidence related to the skeletal and dentoalveolar effects of using skeletal anchorage for maximizing the skeletal effect via applying the forces directly or indirectly to the underlying bone of both jaws.

### Objectives

The aim of this systematic review is to assess the available evidence regarding the skeletal and dentoalveolar effects of bi-maxillary skeletal anchorage devices (BMSADs) used in treating growing class II malocclusion patients.

## Methods

### Protocol registration

The study protocol was registered at the International Prospective Register of Systematic Reviews (PROSPERO) (registration number: CRD42020199601) and was conducted according to the guidelines of the Cochrane Oral Health Group’s Handbook for Systematic Reviews of Interventions (http://ohg.cochrane.org).

### PICOS question and eligibility criteria

Table 1 shows the PICOS (Population, Intervention, Comparison, Outcome and Study design) question, along with the inclusion and exclusion criteria. In brief, the included studies were longitudinal (retrospective or prospective follow-up observational studies) and controlled and non-controlled clinical trials evaluating treatment/observational changes in cephalometric skeletal, dentoalveolar, and occlusal measurements (outcomes) after treatment with BMSADs. The BMSADs consisted of miniscrews and/or miniplates positioned on both jaws (intervention) in growing patients with skeletal Class II malocclusion (population). The studies compared between treated and untreated groups. Studies were excluded if they were case report, case series, literature reviews, systematic review, opinion article, or book chapter, as were studies on patients with craniofacial anomalies, transverse discrepancies, and skeletal asymmetries.

**Table 1:** PICOS question and inclusion and exclusion criteria and search keywords used for the study selection

Category	Inclusion criteria	Exclusion criteria
Participants	Growing patients (patients near the pubertal growth spurt as determined by the cervical vertebral maturation index) with skeletal class II malocclusion or skeletal class II or Angle class II or mandibular retrusion or mandibular hypoplasia or mandibular retrognathism	Patients with craniofacial anomalies and/or transverse discrepancies and/or skeletal asymmetries
Intervention	Orthopedic or interceptive or early treatment using bi-maxillary skeletal anchorage or bone anchor or miniscrew or miniplate or mini-implant or bone screw or bone plate	Single jaw skeletal anchorage device
Comparator	Either control group with no treatment or comparison with other devices	Studies with no control group
Outcome	Primary outcome: skeletal change Secondary outcomes: dentoalveolar changes	Outcomes other than skeletal and dentoalveolar changes
Study Design	Longitudinal (Retrospective or prospective) studies, and controlled and non-controlled clinical trials	Case reports, case series, literature reviews, systematic review, opinion articles, book chapters

### Information sources, search strategy, and study selection

Four co-authors performed an independent comprehensive search on the following six search engines/databases: PubMed, Scopus, Science Direct, Web of Science, Cochrane, and LILACS. The search was later on augmented with a manual search of the reference lists of the included studies. The search was performed twice (once in August 2020 and updated in November 2021). The search keywords of each component of the PICOS question are listed in Table 1.

Duplicates were removed. The titles and abstracts of the remaining articles were screened for potential inclusion, and irrelevant studies were excluded. The full texts of the remaining articles were thoroughly read, and irrelevant studies were removed. This procedure was done independently by two co-authors. Next, the potentially included studies were independently assessed by all co-authors to further confirm whether they met all listed inclusion and exclusion criteria. Disagreements, if any, were resolved via discussion to reach a final consensus. This systematic review was reported according to the Preferred Reporting Items for Systematic Reviews and Meta-Analyses (PRISMA) statement [38].

### Data collection

Data extraction was performed independently by two co-authors, and if any doubts occurred, they were discussed with a third co-author. The data extraction procedure followed a pre-designed template. The following qualitative and quantitative information was extracted: year of publication; study design; inclusion and exclusion criteria; number, gender, and age of patients; type of appliance used for intervention or comparison; skeletal maturational age; site and number of miniscrews/miniplates inserted; means of attachment (direct or indirect); dimensions of the fixing screws (diameter × length in mm); applied force (g); method of outcome assessment (2D/3D); measurements used; follow-up time; treatment duration; and conclusion. The reported treatment effects were the treatment changes, which were measured as the differences between the pre- and post-cephalometric measurements in the interventional and comparison/observational groups separately.

### Outcome assessment

The main skeletal and dentoalveolar outcomes assessed are listed in Table 2.

**Table 2:** The skeletal and dentoalveolar parameters evaluated in the systematic review

Parameter	Abbreviation and/or unit	Definition
Maxillary base position	SNA°	The angle between 3 point landmarks S, N and A point, determining the anteroposterior position of the maxilla relative to the cranial base
Mandibular base position	SNB°	The angle between 3 point landmarks S, N and B point, determining the anteroposterior position of the mandible relative to the cranial base
Sagittal skeletal relation	ANB°	The angle between 3 point landmarks, A point, N and B point, determining the anteroposterior relation between maxilla and the mandible relative to the cranium
Vertical skeletal relation	MPA = SN/Go-Me* or SN/Go-Gn°	The angle between the line S–N and the mandibular plane, measuring the mandibular base tipping relative to the cranium
Maxillary incisors inclination	U1/PP Or U1/SN° or	The angle formed between the palatal plane or SN line and the long axis of the most protruded maxillary incisor
	U1-VP mm	The linear distance between U1 and the Vertical plane
Mandibular incisors inclination	IMPAº	The angle formed between the mandibular plane and the long axis of the most protruded mandibular incisor
Overjet	mm	The horizontal distance between the palatal surface of the most protruded maxillary incisor and the labial surface of the most protruded mandibular incisor
Overbite	mm	The vertical distance between the incisal edge of the most protruded maxillary incisor and the incisal edge of the most protruded mandibular incisor

### Risk of bias

The risk of bias was assessed independently by three co-authors by following a modified checklist from previous publications for the appraisal of randomized and non-randomized studies [39, 40]. Disagreements, if any, were resolved via discussion to reach a consensus. The checklist comprised a total of 11 items: 6 items addressing the study design (type of study, consecutive cases, sample size, control group, selection criteria, and sample size calculation); 2 items addressing the methodological soundness (force magnitude and outcome measures); and 3 items addressing the data analysis (error of the method, statistical analysis, and data presentation). The maximum score was 24. The studies were evaluated as having low, medium, and high levels of evidence if their scores were less than 13, 14–21, and 22–24, respectively. More details are presented in Table 3.

**Table 3:** Quality assessment tool of the included studies

Author (year) [references]	Study design						Methodological		Data analysis			
	Type of study	Consecutive cases (1 )	Sample size (2)	Control group (2)	Selection criteria (2)	Sample size calculation (1)	Outcome measure (4)	Force magnitude (2)	Error of the method (2)	Statistical analysis (2)	Data presentation (2)	Total (24)
Ozbilek et al. [32]	2	0	1	1	1	1	4	1	2	2	2	17
Al-Dumaini et al. [7]	2	0	2	2	2	1	4	1	2	2	2	20
Manni et al. [33]	2	1	2	1	2	0	4	0	2	2	2	18
Kochar et al. [34]	4	0	2	2	2	1	4	0	2	2	2	21

1. Type of study: 0, if retrospective study; 2, if prospective study; 4, if randomized controlled clinical trial.

2. Consecutive cases: 0, if sample comprised inconsecutive patients or if no information regarding this was provided; 1, if sample comprised consecutive patients.

3. Sample size: 0, if ≤ 10 subjects; 1, if > 10 and ≤ 20 subjects; 2, if > 20 subjects.

4. Control group: 0, if no control; 1, if active control; 2, if inactive control

5. Selection criteria: 0, if no cephalometric or dental criteria reported; 1, if cephalometric or dental criteria reported; 2, if cephalometric and dental criteria reported.

6. Sample size calculation: 0, no sample size calculation; 1, sample size calculation.

7. Outcome measure: 0, no values reported; 1, anteroposterior or vertical cephalometric measurement reported; 2, anteroposterior and vertical skeletal cephalometric measurement reported; 4 anteroposterior, vertical cephalometric and dental measurement reported.

8. Force magnitude: 0, if not stated; 1, if stated; 2, if controlled by a force measurement device.

9. Error of the method: 0, if method error not evaluated; 1, if partially adequate method error analysis; 2, if adequate method error.

10. Statistical analysis: 0, if inadequate; 2, if adequate.

11. Data presentation: 0, if inadequate; 1, if P value stated; 2, if any variability measures stated (standard deviation, confidence interval, or range).

From 0 to 13 points: low level of evidence; from 14 to 21 points: medium level of evidence; from 22 to 24 points: high level of evidence

The treatment changes in skeletal, dentoalveolar, and occlusal cephalometric outcomes were retrieved for each individual group (interventional and comparison/observational). Due to variability in these measurements among the included studies, the most commonly used measurements describing the following were obtained: maxillary base position, mandibular base position, sagittal skeletal relation, vertical skeletal relation, maxillary incisors inclination, mandibular incisors inclination, overjet, and overbite.

### Statistical analyses

There was substantial heterogeneity amongst the included studies, which precluded conducting meta-analyses.

## Results

### Study selection

**Fig. 1:**
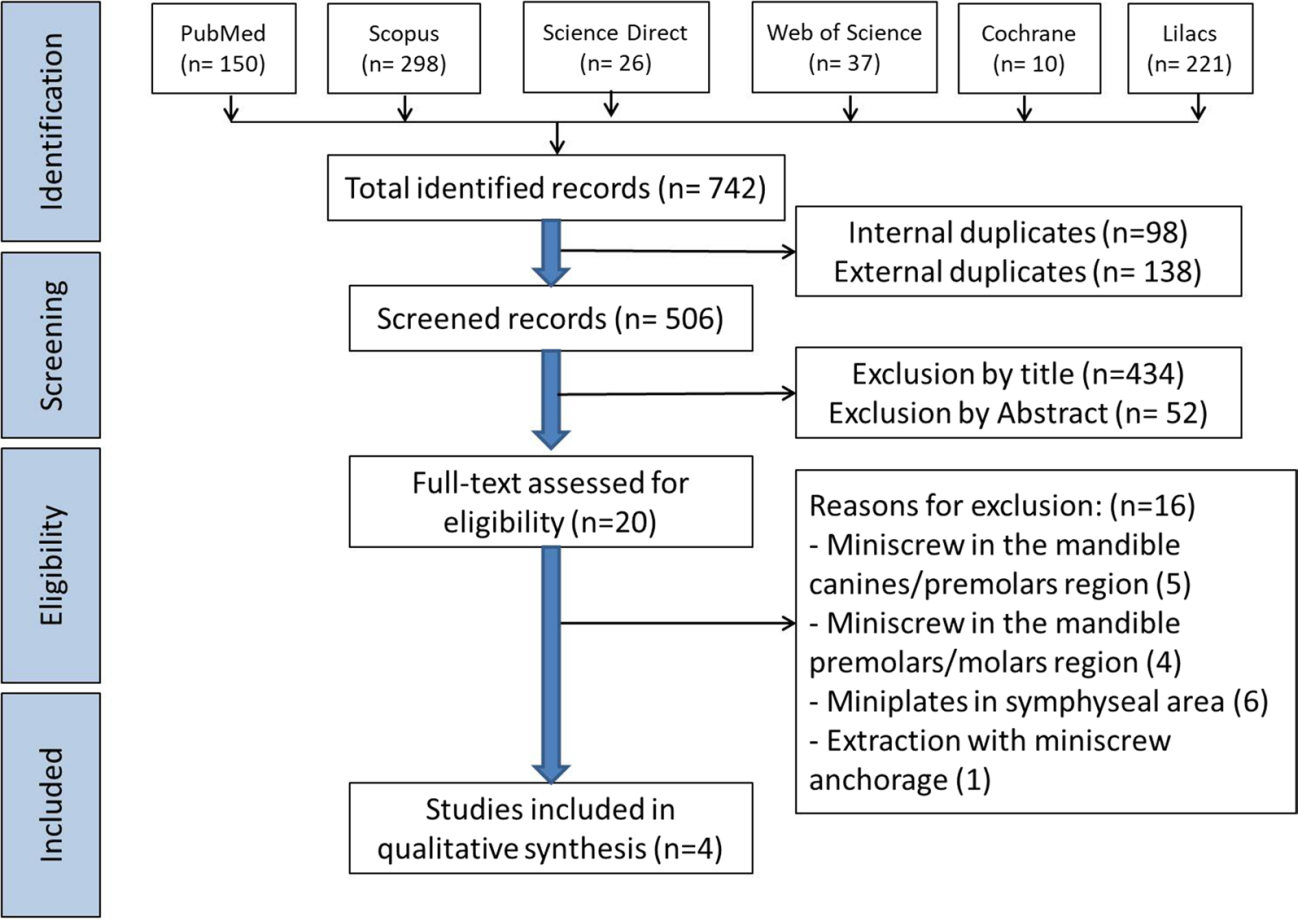
PRISMA diagram of article retrieval

The PRISMA [41] flow chart (Fig. 1) presents the results of the search process. A total of 742 studies were retrieved, of which 236 were excluded as duplicates. After screening the remaining 506 studies by titles and abstracts, 486 were excluded due to irrelevance to the review question. The full texts of the remaining 20 studies were thoroughly read, and 16 were excluded: 5 studies [28–30, 42, 43] used single-jaw miniscrews in the mandible canine/premolar region, 4 studies [44–47] placed miniscrews in the mandible premolar/molar region only, 6 studies [31, 48–52] placed miniplates in the symphyseal area, and one study [53] used miniscrews for anchorage.

### Quality assessment

Table 3 presents the risk of bias assessment for the included studies. All studies [7, 32–34] showed a moderate overall risk of bias. This was mainly due to a lack of randomization, which was only performed in one study, as well as a lack of reporting of the consecutive nature of the sample selection, unlike the case in the study by Manni et al.[33]. The control group was inactive controls (observation/untreated) in the studies by Al-Dumaini et al.[7] and Kochar et al.[34], while the other studies compared BMSADs with active controls using either the same appliance [33] or a different appliance [32]. The substantial heterogeneity amongst the included studies precluded conducting meta-analyses.

### Characteristics of the participants and interventions

Table 4 shows a detailed overview of the studies’ characteristics, procedures, and patient demographics. All four studies were prospective in design. The inclusion and exclusion criteria were similar between studies. The included patients presented with skeletal class II malocclusion in a growing patient with mandibular deficiency, average or horizontal growth pattern, at least half unit class II malocclusion, and ANB > 4°.

**Table 4:** Summary of the studies included in the qualitative analysis

Author (year) [references]	Study design	Inclusion criteria	No. of patients/study/ Control /dropout	Gender (study), (Co.)/age (mean ± SD) Study/Co	Type of appliance (study)/(co.)	Skeletal maturational age	Site and number of miniscrew/ miniplate insertion	Mean of attachment (direct, indirect) study/co./
Ozbilek et al. [32]	Prospective study	(1) Full Class II molar relationship, (2) minimum 5 mm overjet, (3) horizontal or normal growth pattern, (4) minimal crowding, (5) no extracted or missing permanent teeth (excluding third molars)	15 patients/ Study 6 / Control 6 / Dropout 3	Study (3 boys, 3 girls), Control (3 boys, 3girls) Study (12.9 ± 1.5 years), Control (12.3 ± 1.6 years)	Study: miniplates anchored Class II elastics Control: monobloc appliance	In an active growth period (peak stage of pubertal growth determined according to the methods of Bjork, and Grave and Brown) MP3cap of the middle finger	Two miniplates were placed bilaterally at the ramus of the mandible and another two miniplates were placed at the aperture piriformis area of the maxilla	Study: direct control: monobloc appliance
Al-Dumaini et al. [7]	Prospective study	(1) 10 to 13 years; (2) ANB ≥ 5°); (3) deficient mandible; (4) NAPg ≥ 190; (5) average or vertical pattern of growth; (6) ≥ 1/2 Class II molar and canine; (7) overjet ≥ 5 mm	54 patients /Study 28/Control 24/Dropout 2	Study (14 boys, 14 girls), Control (11 boys, 13 girls) Study (11.83 ± 0.85 years), Control (11.75 ± 0.75 years)	Study: miniplates Control: No treatment	Before the pubertal growth spurt (according to cervical vertebrae maturational index)	Two miniplates were placed bilaterally in the posterior buccal area above the external oblique ridge and another two miniplates were placed at the aperture piriformis area of the maxilla	Study: direct control: no treatment
Manni et al. [33]	Prospective study	(1) Class II skeletal relationships (ANB ≥ 4◦), (2) overjet ≥ 4 mm, (3) bilateral Class II molar relationships ≥ half a cusp	26 patients / Study 13 /Control 13 / Dropout (-)	Study (10 boys, 3 girls), Control (3 boys,10 girls) Study (12.8 ± 1.5 years), Control (12.2 ± 1.3 years)	Study: skeletally anchored Herbst appliance with miniscrews Control: standard Herbst appliance without miniscrews	Patients near the pubertal growth spurt (determined by the cervical vertebral maturation [CVM] method; stage CVM 3)	In the maxillary and mandibular arch, miniscrews were placed between the mandibular first and second premolars or between the second premolars and the first molars in the attached gingiva depending on the subject's anatomy	Study: indirect control: no miniscrew
Kochar et al. [34]	Prospective study	(1) Skeletal class II malocclusion due to mandibular retrognathism, (2) Angle class II division 1 malocclusion, (3) positive visualized treatment objective (VTO), (4) overjet greater than 6 mm, (5) average or horizontal growth pattern, (6) minimal crowding (< 3 mm) in both arches	32 patients / Study 16 / Control 16 / Dropout (-)	Study (8 boys, 8 girls), Control (9 boys,7 girls) Study (12.37 ± 1.09 years), Control (12.06 ± 1.34 years)	Study: bimaxillary skeletal anchorage supported fixed function appliance Control: No treatment	Peak of pubertal growth spurt (determined by the cervical vertebral maturation [CVM] method; stage CVM 3)	In the maxilla, “L” shaped bone plates were placed 4–5 mm above the apices of maxillary first molar and in the mandible “T” shaped plates were positioned along the mandibular canine	Study: direct control: no treatment

Author (year) [references]	Screw dimensions (diameter × length mm)	Force (g)	Method of assessment (2D/3D)	Measurements used	Treatment duration	Conclusion
Ozbilek et al. [32]	2 mm diameter × 7 mm length	500 g	2D (lateral cephalometry)	*Angular measurements*: SNA, SNB, ANB, angle of convexity, SN-Go-Gn, SN-PP, SN-OP, FMA, U1-PP, IMPA, U1-L1 *Linear measurements* Co-A, A-VRL, A-HRL, Co-Gn, B-VRL, B-HRL, Pog-VRL, Pog-HRL, Witts, U1-VRL, L1-VRL, overjet, overbite, Ls-VRL, Li-VRL, Pog(s)- VRL	Mean duration time was 0.68 ± 0.05 years for the elastics group and 0.65 ± 0.09 years for the monobloc group	1- Effective mandibular length was significantly greater in the miniplate-anchored group 2- Mandibular incisor retrusion was observed in the miniplate-anchored, whereas mandibular incisor protrusion was found in the monobloc group
Al-Dumaini et al. [7]	2 mm diameter × 6 mm length	450 g 250 g per side for the first 3 weeks followed by 350 g per side for the next 3 weeks to reach 450 g per side for the rest of the functional treatment phase	2D (lateral cephalometry)	*Angular measurements*SNA, SNB, ANB, SNPog, NAPog, ArGoMe, SN-PP, SN-GoMe, U1-SN, L1-GoMe, U1-L1 A-Y axis, Ar-Go, Go-Me, Co-Go, Co-Pog, Pog-Y axis, B-Y axis, S-Go, N-Me, overjet, overbite	The initial alignment and leveling phase lasted for an average of 7 months followed by a functional phase for an average of 9 months	Bimaxillary miniplates-based skeletal anchorage results to increase in mandibular ramal and body lengths and counter-clockwise mandibular positioning
Manni et al. [33]	1.4 diameter mm × 8 mm length	Not reported	2D (lateral cephalometry)	*Angular measurements* SNA, SNB, ANB, SN/GoGn, Is/PP, Ii/GoGn, *Linear measurements* Wits, A-OLp, Pg-OLp, Co-OLp, Is-OLp, Ii-OLp, Overjet, Ms-OLp, Mi-OLp	The mean treatment times were 10.0 ± 0.8 months in the treatment group and 10.8 ± 2.1 months in the control group	1- Anchorage reinforcement using miniscrews reduced flaring of the mandibular incisor 2- The upper molars showed a slightly forward movement in HA with miniscrew anchorage
Kochar et al. [34]	2 mm diameter × 7 mm length	Not reported	2D (lateral cephalometry)	*Angular measurements* SNA, SN-Pal Pl, NA-Pal Pl, SNB, FMA, SN-GoGn, IMPA, ANB, NA-Pog, interincisal angle *Linear measurements* A-VP, N-ANS (perpendicular to HP), N-PNS (perpendicular to HP), U1-VP, U1-HP, U6-VP, U6-HP, B-VP, Pog-VP, Co-Gn, Go-Pog, L1-VP, L1-GoMe, L6-VP, L6-GoMe, overjet, overbite	The mean treatment time was 7.44 ± 1.06 months	Bimaxillary skeletal anchorage supported fixed functional appliance showed significant skeletal changes. The changes in the maxilla included retrusion and restricted posterior vertical growth. Mandibular changes included significantly increased mandibular growth with negligible effect on growth pattern

Three studies [7, 33, 34] used the cervical vertebral maturation method to assess the skeletal maturation, while one study [32] used the MP3cap (capping of the epiphysis on the diaphysis of the medial phalanx of the middle finger). Three studies [7, 32, 34] used bi-maxillary miniplates as direct anchorage, and one study [33] used a miniscrew as indirect anchorage. With regard to the control group, Al-Dumaini et al.[7] and Kochar et al.[34] included untreated patients, while Ozbilek et al.[32] treated their control groups with monobloc, and Manni et al.[33] treated them with a standard Herbst appliance without miniscrews. The treatment effects were measured as changes upon comparing pre- and post-treatment/observational two-dimensional lateral cephalograms.

A total of 127 participants were enrolled and 5 patients dropped out. Of the remaining 122 patients, 63 were enrolled in the study group (35 boys and 28 girls), and 59 patients were enrolled as controls (26 boys and 33 girls). The mean age of the study and control groups ranged from 11.83 ± 0.8 [7] to 12.8 ± 1.5 years [33] and from 11.75 ± 0.75 [7] to 12.3 ± 1.6 years [32], respectively. Two studies [7, 34] calculated the sample size in advance. Two of the included studies [7, 32] used removable inter-maxillary protracting force, while the other two used a fixed method [33, 34]. Three studies used miniplates [7, 32, 34], while the fourth one [33] used a miniscrew to support the used fixed functional appliance. Two studies explicitly reported the force levels as 450 g [7] and 500 g [32], while the other two studies did not report this parameter. The force level was the maximum applied force, whether starting on day one of treatment [32] or achieved gradually [7].

The main skeletal evaluation parameters in this review were changes in maxillary base position (SNA), mandibular base position (SNB), and sagittal (ANB) and vertical (MPA) jaw relations [7, 32–34]. The dentoalveolar parameters assessed were changes in maxillary incisor inclination [7, 32, 33] or position [34] and mandibular incisor inclination [7, 32–34]. The occlusal parameters extracted were the changes in the overjet and overbite [7, 32, 34]. Ultimately, four studies [7, 32–34] fulfilled the inclusion criteria and were processed for the subsequent qualitative analysis.

### Characteristics of outcome measures

The reported skeletal, dentoalveolar and occlusal outcomes reported by the included studies could be classified under eight categories: (1) maxillary skeletal position, (2) mandibular skeletal position, (3) sagittal skeletal jaw relation, (4) vertical skeletal jaw relation, (5) maxillary incisor inclination, (6) mandibular incisor inclination, (7) overjet, and (8) overbite.

### Skeletal changes: maxillary and mandibular bases

Table 5 summarizes the results of the skeletal effects of the BMSADs. The mean change in the maxillary base was evaluated by SNA angle, and all studies reported maxillary base retrusion ranging from as low as 0.083** ± **0.96° [32] to as high as 1.40** ± **1.84° [7]. In contrast to the comparison/observation control groups, the reported retrusion was statistically significant in two studies [7, 34]. Based on the SNB angle, all studies reported mandibular advancement ranging from as low as 2.9** ± **1.03/1.8° [7, 33] to as high as 3.25** ± **0.89° [32].

**Table 5:** Summary of mean skeletal changes in the treatment and comparison/observation control groups in the included studies

Author (year) [references]	Maxillary base position (SNA°)			Mandibular base position (SNB°)			Sagittal skeletal relation (ANB°)			Vertical skeletal relation (MPA = SN/Go− Me* or SN/Go− Gn°)		
	Study	Control	*P* value	Study	Control	*P* value	Study	Control	*P* value	Study	Control	*P* value
Ozbilek et al. [32]	− 0.083 ± 0.96	− 0.65** ± **0.27	0.180	3.25± 0.89	2.40 ± 0.90	0.093	− 3.18± 0.84	− 3.20 ± 0.85	0.937	0.83 ± 1.57	1.21 ± 0.49	0.589
Al− Dumaini et al. [7]	− 1.40± 1.84	0.25 ± 0.65	< 0.001	2.9 ± 1.03	0.55**± **0.97	< 0.001	− 4.00± 0.80	− 0.31 ± 1.01	< 0.001	− 2.25 ± 0.95*	0.50 ± 1.00	< 0.001
Manni et al. [33]	− 0.7 ± 1.6	− 1.0** ± **2.1	0.62	2.9± 1.8	1.1 ± 2.8	0.02	− 3.3± 1.8	− 1.3± 1.3	0.01	− 0.5 ± 2.1	2.2 ± 2.7	0.01
Kochar et al. [34]	− 1.29± 0.59	0.18** ± **0.39	< 0.001	3± 0.87	0.29 ± 0.47	< 0.001	− 4.2± 0.99	− 0.17 ± 0.64	< 0.001	0.41 ± 0.51	0.59 ± 0.51	0.27

SNA°: The angle between 3 point landmarks S, N and A point, determining the anteroposterior position of the maxilla relative to the cranial base

SNB°: The angle between 3 point landmarks S, N and B point, determining the anteroposterior position of the mandible relative to the cranial base

ANB°: The angle between 3 point landmarks, A point, N and B point, determining the anteroposterior relation between maxilla and the mandible relative to the cranium

MPA°: The angle between the line S–N and the mandibular plane, measuring the mandibular base tipping relative to the cranium

In contrast to the comparison/observation control groups, the reported mandibular advancement was statistically significant in all studies except one [32]. Similarly, the anteroposterior jaw relation measured by ANB angle was reported to be reduced with a range of 3.18 ± 0.84° [32] to 4.29 ± 0.99° [34]. The vertical jaw relation was evaluated by SN/Go-Gn in three studies [32–34] and by SN/Go-Me in one study [7]. Two studies recorded statistically insignificant mean increases of the mandibular plan angle (MPA) of 0.83 ± 1.57° [32] and 0.41 ± 0.51° [34], while the other two studies reported statistically significant mean decreases of 2.25 ± 0.95° [34] and 0.5 ± 2.1° [33].

### Dentoalveolar changes: upper and lower incisors (U1, L1)

Table 6 shows the results of the effect of the BMSADs on the maxillary and mandibular incisors inclination and/or position. The measurement methods used for the maxillary incisor inclination in the included studies were variable. Ozbilek et al. [32] and Manni et al. [33] evaluated this inclination relative to the palatal plan, and Al-Dumaini et al. [7] used the SN plan as a reference. Kochar et al. [34] measured the horizontal distance between the vertical plan and the maxillary incisors. Ozbilek et al. [32] and Manni et al. [33] reported increases in the maxillary incisor inclination by 4.6 ± 2.40° (statistically significant relative to the comparison control group) and 5.1 ± 7.7° (statistically insignificant relative to the comparison control group), respectively.

**Table 6:** Summary of dentoalveolar changes in the treatment and/ comparison/observation control groups in the included studies

Author (year) [references]	Maxillary incisors inclination (U1/PP Or U1/SN° or U1− VP mm)			Mandibular incisors inclination (IMPA°)			Overjet (mm)			Overbite (mm)		
	Study	Control	*P* value	Study	Control	*P* value	Study	Control	*P* value	Study	Control	*P* value
Ozbilek et al. [32]	4.60 ± 2.40	− 2.33 ± 1.87	0.002	− 3.01± 1.66	5.45± 1.23	0.002	− 4.80 ± 1.18	− 3.81 ± 0.67	0.180	− 2.53 ± 1.31	− 3.55 ± 0.48	0.240
Al− Dumaini et al. [7]	− 1.15 ± 0.94	0.40 ± 0.97	< 0.001	− 1.27 ± 2.48	0.47 ± 1.58	0.002	− 4.26 ± 0.85	− 0.12 ± 0.44	< 0.001	1.47 ± 0.73	− 0.13 ± 0.23	< 0.001
Manni et al. [33]	5.1 ± 7.7	1.0 ± 9.4	0.33	1.6 ± 5.6	3.7 ± 4.2	0.40	− 3.7 ± 2.6	− 3.8 ± 1.9	0.44	−	−	−
Kochar et al. [34]	0.24 ± 0.44	0.35 ± 0.49	0.33	3.35 ± 0.86	0.53 ± 0.51	< 0.001	− 5.44 ± 1.26	− 0.38 ± 0.62	< 0.001	− 3.69 ± 0.60	− 0.38 ± 0.50	< 0.001

U1/PP°: The angle formed between the palatal plane and the long axis of the most protruded maxillary incisor

U1/SN°: The angle formed between the cranial base plane and the long axis of the most protruded maxillary incisor

U1-VP (mm): The linear perpendicular distance between the vertical plan and the incisal edge of the most protruded maxillary incisor

IMPA°: The angle formed between the mandibular plane and the long axis of the most protruded mandibular incisor

In contrast, relative to the observational control groups, Al-Dumaini et al. [7] recorded a statistically significant decrease in the maxillary incisors inclination by 1.15** ± **0.94°, while Kochar et al. [34] found a statistically insignificant positive change in the maxillary incisors position by 0.24 ± 0.44 mm. The inclination of the mandibular incisors was measured relative to the mandibular plan in all included studies. Two studies reported proclination of the mandibular incisors by 3.35** ± **0.86° [34] (statistically significant relative to the comparison control group) and 1.6 ± 5.6° [33] (statistically insignificant relative to the comparison control group), while the other two studies reported statistically significant retroclination by 1.27 ± 2.48° [7] and 3.01** ± **1.66° [32] relative to the observational control groups.

### Occlusal parameters: overjet and overbite

The overjet was reported to be reduced in all included studies. Relative to the comparison/observational groups, the reduction in the BMSAD groups was statistically significant according to Al-Dumaini et al. [7] and Kochar et al. [34] at 4.26 ± 0.85 and 5.44 ± 1.26 mm, respectively. In contrast, Ozbilek et al. [32] and Manni et al. [33] reported statistically insignificant reductions in overjet relative to the comparison/observational groups of 4.80 ± 1.18 and 3.7 ± 2.6 mm, respectively. Two studies [32, 34] reported reduction in the overbite by 2.53 ± 1.31 mm (statistically insignificant relative to the control group) and 3.69 ± 0.60 mm (statistically significant relative to the control group). Conversely, Al-Dumaini et al. [7] recorded a statistically significant increase in overbite (relative to the control group) by 1.47 ± 0.73 mm. Manni et al. [33] did not measure the overbite in their study.

## Discussion

Based on the limited evidence, and even heterogeneous available literature, BMSADs, more specifically the miniplate-based anchorage class II correctors, were found to produce a significant maxillary skeletal retrusion and mandibular base protrusion and if the applied force is removable like that accompanied with class II elastics, less proclination of the mandibular incisors was reported compared to fixed ones (FFAs).

The use of skeletal anchorage devices in orthodontic and orthopaedic treatment has been gaining popularity among orthodontists. The aim of using miniplates or miniscrews as an anchorage aid is to minimize the need for patients’ cooperation and to maximize the skeletal and dentoalveolar effects in growing patients with malocclusion. Indeed, proclination of the mandibular incisors is a compensating phenomenon in class II malocclusions and occurs with many devices used to correct this common type of malocclusion [54], which represents a major disadvantage.

This systematic review revealed that BMSADs exert a retrusive effect on the maxilla ranging from almost a negligible one (0.083 ± 0.96°) [32] to modest and statistically insignificant effects relative to the active treatment used for the control group (0.7 ± 1.6°) [33]. This can be ascribed to the control groups being active groups treated with monobloc and Herbst FFA, respectively, which are well known to exert retrusive effects on the maxilla. In contrast, the maxillary retrusive effect was obvious and both statistically and clinically significant in the remaining two studies (1.40 ± 1.84 and 1.29 ± 0.59°) as compared to the observational (untreated) controls [7, 34]. It seems that miniplate-supported appliances have a retrusive maxillary effect due to the proximity of the force applied to the centre of resistance of the maxilla.

Regarding the treatment effect on the mandibular base, this systematic review showed that BMSADs had a statistically and clinically significant protrusive effect relative to the comparison/observational control groups in all included studies except for one [32]. The insignificant effect reported by Ozbilek et al. [32] must be considered with caution as they treated the patients in the comparison control group with a removable functional appliance (monobloc), which is reported to have a statistically and clinically significant mandibular advancing effect [20]. However, the mandibular advancing effect of BMSADs was obvious in comparison with the Herbst functional appliance, as shown by Kochar et al. [34]. Collectively, BMSADs using either miniplates or miniscrews produce sagittal mandibular advancement, regardless of the inter-maxillary protracting force.

The present systematic review found that the vertical jaw relation, represented by the mandibular plan angle, was either significantly reduced or insignificantly increased. Reduction is the preferable effect in most of the cases of targeted class II division 1 malocclusion, which makes BMSADs a promising approach. The highest reduction was reported by Al-Dumaini et al. [7] at 2.25 ± 0.95° in the study group compared to 0.50 ± 1.00° in the observational control group. Such a reduction can be ascribed to the proper position of the mandibular miniplates relative to the centre of resistance of the mandible, making the counter clockwise mandibular rotation biomechanically more possible.

In contrast, Manni et al. [33] reported an almost neglected reduction in the vertical jaw relation by 0.5 ± 2.1° in the study group (Herbst FFA with miniscrews) compared to the significant increase by 2.2 ± 2.7° that happened in the control group (Herbst FFA). Thus, miniscrew-supported FFAs seem to control the forward movement of the mandibular incisors, which might be responsible for opening the bite and increasing the vertical jaw relation.

Al-Dumaini et al. [7] and Ozbilek et al. [32] applied the same concept: bimaxillary miniplates with intermaxillary elastics. However, the positions of the mandibular miniplates differed: Al-Dumaini et al. [7] used the buccal oblique ridge, which is closer to the centre of resistance of the mandible, while Ozbilek et al. [32] used a position that was more posteriosuperior in the anterior border of the ramus. Such positioning relative to the centre of resistance of the mandible might explain the opposite findings of both studies. In the study by Ozbilek et al. [32], the insignificantly higher MPA that was reported in the monobloc group compared to the miniplates group can be attributed to the extrusion of the mandibular posterior teeth in the monobloc group [20]. It seems that BMSADs maintain or even improve the vertical jaw relation under two situations: firstly when miniplates/miniscrews are positioned close to the centre of resistance of the mandible, and secondly when the inclination of the mandibular incisors is controlled.

In contrast to the favourable retrusive effect on the maxillary incisors (− 1.15 ± 0.94° vs. 0.40 ± 0.97) reported by Al-Dumaini et al. [7], the present systematic review shows that BMSADs exert an unfavourable proclination effect on the maxillary incisors, which was seen more prominently by Manni et al.[33] (5.1 ± 7.7° vs. 1.0 ± 9.4°) and Ozbilek et al. [32] (4.60 ± 2.40° vs. − 2.33 ± 1.87°). The protrusive effect in the former study can be explained by the force exerted by the clear aligner appliance on the mandibular arch, which was primarily placed to control the proclination of the mandibular incisors. The protrusive effect in the latter study can be ascribed to the contact of the labial surfaces of the mandibular incisors with the palatal surfaces of the maxillary incisors as the mandible moves forward under the influence of the intermaxillary elastic forces. The favourable retroclination effect in the monobloc control group (2.33 ± 1.87°) in the study by Ozbilek et al. [32] is explained by the reciprocal effect of the appliance on the maxillary incisors aided by the labial bow.

Inclination of the mandibular incisors is a critical factor during orthopaedic treatment of class II malocclusion. Most of the published systematic reviews report a proclination effect of both removable appliances and FFAs as a compensating or camouflage effect [20, 25–27]. Based on the mechanics used, BMSADs can result in an interesting and favourable retroclination of the mandibular incisors. For example, Al-Dumaini et al. [7] and Ozbilek et al. [32] applied similar mechanics: they used miniplates and intermaxillary class II elastics. Ozbilek et al. [32] reported significant retroclination of mandibular incisors in the BMSADs (− 3.01 ± 1.66°) compared to proclination in the monobloc group (5.45 ± 1.23°), while Al-Dumaini et al.[7] found  a significant retroclination in the mandibular incisors (− 1.27 ± 2.48°) in the miniplates group compared to proclination (0.47 ± 1.58°) in the observational controls.

However, the BMSAD approach used by Kochar et al. [34] resulted in what is called a “class III effect” on the mandibular incisors: there was a statistically and clinically significant unfavourable proclination of the mandibular incisors in the BMSAD group (3.35 ± 0.86°) compared to the observational controls (0.53 ± 0.51°). The fourth study found insignificant proclination effects on the mandibular incisors [33].

Celikoglu et al. [51] and von Bremen et al. [44] reported a favourable retroclination of mandibular incisors when using mandibular skeletal anchor-supported FFA, while Aslan et al. [28] reported unfavourable proclination of the mandibular incisors (3.06°). Whenever skeletal anchorage devices are planned, the effectiveness of miniplates over miniscrews for controlling inclination of the mandibular incisors is a pivotal factor to consider. Up to 85% of the class II correction with an FFA occurs due to the proclination of the mandibular incisors, which is an undesirable side effect that must be addressed properly to achieve optimum treatment results. The BMSAD approach seems to be a reasonable and promising solution.

This systematic review showed that BMSADs reduced the overjet by 3.7 ± 2.6 [33] to 5.44 ± 1.26 mm [34] and reduced the deep overbite by 2.53 ± 1.31 [32] to 3.69 ± 0.60 mm [34]. However, there was an exception for Al-Dumaini et al. [7], who reported increased overbite (1.47 ± 0.73 mm). This result can be attributed to the simultaneous retroclination of both maxillary and mandibular incisors.

### Limitations

In addition to the small number of studies included, another limitation is the moderate overall quality of these studies. Meta-analysis was not possible due to considerable variations among the included studies with regard to study design and assessment methodology. Another limitation was that only English articles were included. Hence, the reported treatment effects of BSSADs should be interpreted with high caution. Caution is also advised when extrapolating the results of this review to patients of different ethnicities. Moreover, standardization regarding the gender, participant characteristics, and skeletal age assessment is advised for future clinical trials and/or observational studies. Additional well-designed, high-quality, randomized controlled trials are required to investigate the efficiency of BMSADs to prove or reject their superiority over conventional methods.

## Conclusions

Keeping in mind the limitations of this review, the following can be concluded:

- 1. The most significant skeletal effect of the miniplate-based anchorage class II correctors was mandibular base protrusion with minimal effect on the maxillary arch.
- 2. At the level of dentoalveolar effects, the use of fixed appliances supported by bi-maxillary anchorage did not control the proclination of the mandibular incisors.
- 3. Combined maxillary and mandibular anchorage improved the occlusal parameters.

The currently available evidence is insufficient to form a sound conclusion regarding the effects of BMSADs in treating growing skeletal class II malocclusion patients.

## Data Availability

The datasets used and/or analyzed during the current study are available from the corresponding author on reasonable request.

## Abbreviations

BMSADs: Bi-maxillary skeletal anchorage devices
FFAs: Fixed functional appliances
